# Uncertainty of adolescent brain maturation sex difference claims

**DOI:** 10.1073/pnas.2420724121

**Published:** 2024-11-25

**Authors:** Cordelia Fine, Liz Cope, Lucienne Elliott, Chloe A. Matthews, Madison Muscat, Christine Polowyj, Harvey L. Warren

**Affiliations:** ^a^School of Historical and Philosophical Studies, The University of Melbourne, Carlton, VIC 3010, Australia

In their article, Corrigan et al. (1) claim that COVID-19 pandemic lockdown measures gave rise to “unusually accelerated brain maturation in adolescents” and that this acceleration was “much more pronounced” in females than males. Arguing this “indicate[s] greater vulnerability of the female brain, as compared to the male brain, to the lifestyle changes resulting from pandemic lockdowns,” they draw links between accelerated maturation, reduced social interaction, and poorer mental health outcomes in females.

However, the approach taken by the authors, which divided and contrasted pre- and postpandemic samples by sex category, does not offer direct support for any of these claims or interpretations. The authors make no direct statistical comparison of the sexes (see ref. 2). They offer no measure of the proposed mechanism of reduced social interaction (despite lockdown experiences being diverse), the speculated outcome (increased mental health risk), nor any measure of association of either with accelerated brain maturation.

Moreover, the researchers do not account for potential confounding factors affecting lockdown lifestyle that might have been unevenly distributed across their small subsamples or systematically differ due to gender norms (e.g., females may have been prevailed upon more than males to assist with increased domestic and care activities (3) or at disproportionately increased risk of family violence).

Nonetheless, the authors propose that acceleration of a natural maturational process in this mentally healthy sample tracks inherently vulnerable female neurobiology, centering this focus in their article title and press release and describing their reported findings of sex differences as “stunning” and “dramatic” to the press (4).

We would question whether this is an appropriate handling of the considerable scientific uncertainty underlying these proposals. Understanding the sex-biased prevalence of disorders of brain and mind is an important line of inquiry. However, sex category is not a causal mechanism, and sex-related variables, including hormones, neurobiological variables, gendered experiences, traits, abilities, or preferences, are rarely binary (5). By categorizing only by binary sex rather than, for instance, measures of lockdown stress or importance of, or reduction in, social interaction, it is unclear how the authors have advanced understanding of how or whether relations between pandemic experiences, brain maturation in adolescents, and mental health outcomes are shaped by sex and/or gender. Meanwhile, if erroneous, the cost of these interpretations is the reinforcement of gender stereotypes of female (male) emotional (in)vulnerability and social (in)dependence.
